# A Novel Strategy for Inducing the Antitumor Effects of Triterpenoid Compounds: Blocking the Protumoral Functions of Tumor-Associated Macrophages via STAT3 Inhibition

**DOI:** 10.1155/2014/348539

**Published:** 2014-03-11

**Authors:** Yukio Fujiwara, Motohiro Takeya, Yoshihiro Komohara

**Affiliations:** Department of Cell Pathology, Graduate School of Medical Sciences, Faculty of Life Sciences, Kumamoto University, 1-1-1 Honjo, Kumamoto 860-8556, Japan

## Abstract

There are many types of nontumor cells, including leukocytes, fibroblasts, and endothelial cells, in the tumor microenvironment. Among these cells, infiltrating macrophages have recently received attention as novel target cells due to their protumoral functions. Infiltrating macrophages are called tumor-associated macrophages (TAMs). TAMs polarized to the M2 phenotype are involved in tumor development and are associated with a poor clinical prognosis. Therefore, the regulation of TAM activation or M2 polarization is a new strategy for antitumor therapy. We screened natural compounds possessing an inhibitory effect on the M2 polarization of human macrophages. Among 200 purified natural compounds examined, corosolic acid (CA) and oleanolic acid (OA), both are categorized in triterpenoid compounds, inhibited macrophage polarization to M2 phenotype by suppressing STAT3 activation. CA and OA also directly inhibited tumor cell proliferation and sensitized tumor cells to anticancer drugs, such as adriamycin and cisplatin. The *in vivo* experiments showed that CA significantly suppressed subcutaneous tumor development and lung metastasis in a murine sarcoma model. The application of triterpenoid compounds, such as CA and OA, is a potential new anticancer therapy targeting macrophage activation, with synergistic effects with anticancer agents.

## 1. Introduction

Macrophages, first identified to be large phagocytes, play a critical role in innate and adaptive immunity by engulfing bacteria and other microbes and secreting several inflammatory molecules. Macrophages were first thought to be deleterious and inflammatory cells. However, recent studies have found that their functional roles are far more numerous. Macrophages are involved in remodeling/repair, neovascularization, atherosclerosis, and tumor development and are now considered multifunctional cells, more so than immune cells [1]. In tissue remodeling/repair processes, macrophages serve as key players for the resolution of inflammation and the restoration of the tissue integrity/function. The beneficial effects of macrophages are primarily due to the trophic factors they release in the environment, particularly those with effects on parenchymal cells. The wide range of active molecules secreted by macrophages likely explains their wide roles in tissue development, repair, and homeostasis demonstrated in various tissues [2].

Macrophages are broadly classified into classically activated macrophages (M1 macrophages) and alternatively activated macrophages (M2 macrophages), according to their functions. M1 macrophages are potent effecter cells that kill microorganisms and produce primarily proinflammatory cytokines, such as tumor necrosis factor *α* (TNF-*α*), IL-6, and IL-12 [3]. In contrast, M2 macrophages reduce these inflammatory and adaptive Th1 responses by producing anti-inflammatory factors (IL-10, TGF-*β*, and IL-1 receptor antagonist) and promoting angiogenesis, tissue remodeling, and repair [3]. M2 macrophages also exhibit a high expression of several receptors, including class A scavenger receptor (SR-A, CD204), mannose receptor (CD206), hemoglobin scavenger receptor (CD163), dectin-1, and DC-SIGN [4–8]. Macrophages are plastic cells, as they can switch from an activated M1 state back to an M2 state, and vice versa, upon the induction of specific signals [3].

Macrophages infiltrating in cancer tissues are referred to as tumor-associated macrophages (TAMs), which are closely involved in the development of the tumor microenvironment [9–11]. Heterogeneity of phenotypes is observed among TAMs in various malignant tumors, and a significant proportion of TAMs with the M2 phenotype is associated with a worse clinical prognosis and high grade of malignancy [4, 11–15].

We previously demonstrated CD163 to be a useful marker for detecting M2 cells on paraffin-embedded surgical specimens [16]. In several human malignant tumors, a proportion of CD163-positive M2 TAMs are closely involved in tumor cell proliferation and associated with a poor prognosis [17–21]. CD163, a member of the scavenger receptor cysteine-rich protein superfamily, is a receptor for the hemoglobin-haptoglobin (Hb-Hp) complex, TNF-like weak inducer of apoptosis (TWEAK), and porcine reproductive and respiratory syndrome virus [22–25]. CD163 also binds bacteria and induces the production of proinflammatory cytokines [26]. It has been reported that binding of the Hb-Hp complex to CD163-bearing cells elicits potent interleukin-10 secretion and the HO-1 expression [27, 28]. These data indicate that CD163 is actively involved in the anti-inflammatory function of M2 macrophages, although the precise ligand receptor effector pathway has not yet been clarified. These observations suggest that macrophage differentiation (M2 polarization) is correlated with tumor development. Therefore, the regulation of macrophage activation is a potential new strategy for cancer immunotherapy.

Based on this background, we attempted to isolate natural compounds that suppress the M2 polarization of macrophages and identified two triterpenoid compounds, corosolic acid (CA), and oleanolic acid (OA), using newly established screening method. Notably, CA and OA not only inhibited M2 polarization but also suppressed tumor cell proliferation and sensitized tumor cells to anticancer drugs. The antitumor effects of CA were confirmed using a murine sarcoma model.

## 2. Screening of Natural Compounds Inhibiting the CD163 Expression in Human Monocyte-Derived Macrophages (HMDMs)

**Figure 1:**
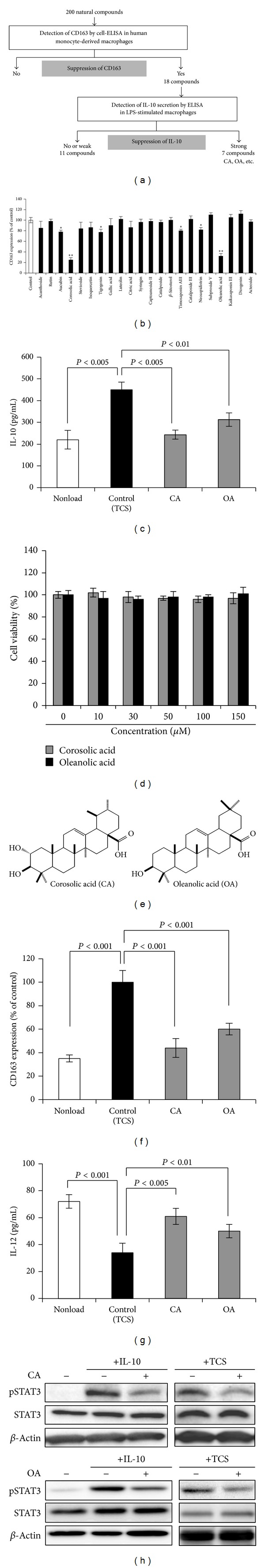
Effects of natural compounds on macrophage activation. A schematic drawing of the compound screening process (a). HMDMs (5 × 10^4^ cells per well in a 96-well plate) were incubated with natural compounds (30 *μ*M) for 24 hours after treatment with IL-10 (20 nM) for two days, after which the CD163 expression was determined using Cell-ELISA (b). HMDMs (5 × 10^4^ cells per well in a 96-well plate) were stimulated with LPS (100 ng/mL) for 24 hours after incubation with 30 *μ*M of corosolic acid (CA) and 30 *μ*M of oleanolic acid (OA) for 24 hours in the presence of TCS, after which the level of IL-10 secretion was determined using ELISA (c). HMDMs (5 × 10^4^ cells per well in a 96-well plate) were incubated with the indicated concentrations of corosolic acid (CA) and oleanolic acid (OA) for 24 hours, after which the cell viability was determined using a WST-8 assay (d). Chemical structures of corosolic acid (CA) and oleanolic acid (OA) (e). HMDMs (5 × 10^4^ cells per well in a 96-well plate) were incubated with 30 *μ*M of corosolic acid (CA) and 30 *μ*M of oleanolic acid (OA) for 24 hours after treatment with U373 glioblastoma-derived tumor cell supernatant (TCS) for two days, after which the CD163 expression was determined using Cell-ELISA (f). HMDMs (5 × 10^4^ cells per well in a 96-well plate) were stimulated with LPS (100 ng/mL) for 24 hours after incubation with 30 *μ*M of corosolic acid (CA) and 30 *μ*M of oleanolic acid (OA) for 24 hours in the presence of TCS, after which the level of IL-12 secretion was determined using ELISA (g). HMDMs were incubated with 30 *μ*M of corosolic acid (CA) or oleanolic acid (OA) for three hours after treatment with IL-10 (20 nM) or TCS for 24 hours, after which the levels of phosphorylated STAT3, STAT3, and *β*-actin were determined using a Western blot analysis (h). The data are presented as the mean ± SD. *P < 0.01, **P < 0.001 versus control.

It is well known that enhancement of the CD163 expression, a useful cell surface marker of M2 phenotype, is accompanied by IL-10-induced M2 macrophage polarization. Recently, we established a Cell-ELISA system to detect the CD163 expression in macrophages in order to screen natural compounds regulating macrophage activation [29]. We first measured the effects of 200 natural compounds (selected compounds having famous bioactive structure such as flavonoid compounds, triterpenoid compounds, and steroid compounds from our natural compound library) on the IL-10-induced CD163 expression in human monocyte-derived macrophages (HMDMs). Some natural compounds, including aucubin, CA, tigogenin, timosaponin AIII, neoaspidistrin, and OA, suppressed the CD163 expression (Figures 1(a) and 1(b)).

IL-10 secretion induced by stimulation with LPS is also used to evaluate M2 polarization. The tumor cell culture supernatant (TCS) of the U373 glioblastoma cell line induces the upregulation of IL-10 secretion from macrophages [29]. Among these compounds, CA and OA, triterpenoid compounds, were found to significantly suppress IL-10 secretion from LPS-stimulated macrophages (Figure 1(c)), whereas CA and OA caused no morphological changes or cytotoxic effects in the HMDMs (Figure 1(d)). Therefore, we chose these triterpenoid compounds for further investigation (Figure 1(e)). Next, we measured the effects of CA and OA on the secretion of IL-12 and the expression of CD163 in HMDMs induced by TCS. Stimulation with TCS increased the CD163 expression (Figure 1(f)) and decreased IL-12 secretion (Figure 1(g)), a M1 phenotype marker, in the HMDMs. Under the employed assay conditions, CA and OA significantly suppressed the TCS-induced CD163 expression (Figure 1(f)) and enhanced the IL-12 secretion reduced by TCS treatment (Figure 1(g)). These data indicate that CA and OA change M2 polarization to M1 polarization in HMDMs and regulate macrophage activation.

## 3. Effects of CA and OA on STAT3 Activation in the HMDMs 

It is well known that signal transducer and activator of transcription 3 (STAT3) is involved in the creation of the tumor microenvironment and tumor development due to its association with immunosuppression, angiogenesis, and cancer cell proliferation [30]. STAT3 signaling in macrophages is also involved in the regulation of immune responses in murine models [31, 32], and STAT3 activation is essential for macrophage differentiation toward the M2 phenotype [33]. Therefore, we next investigated the effects of CA and OA on IL-10- and TCS-induced STAT3 activation in HMDMs. As shown in Figure 1(h), STAT3 phosphorylation was increased in the HMDMs by stimulation with IL-10 and TCS. Under the employed assay conditions, CA and OA significantly inhibited the IL-10-induced STAT3 activation and suppressed the TCS-induced STAT3 activation in the HMDMs (Figure 1(h)). These results suggest that CA and OA change M2 polarization to M1 polarization in HMDMs by suppressing STAT3 activation.

## 4. Effects of CA and OA on STAT3 Activation and Proliferation in Tumor Cells 

**Figure 2:**
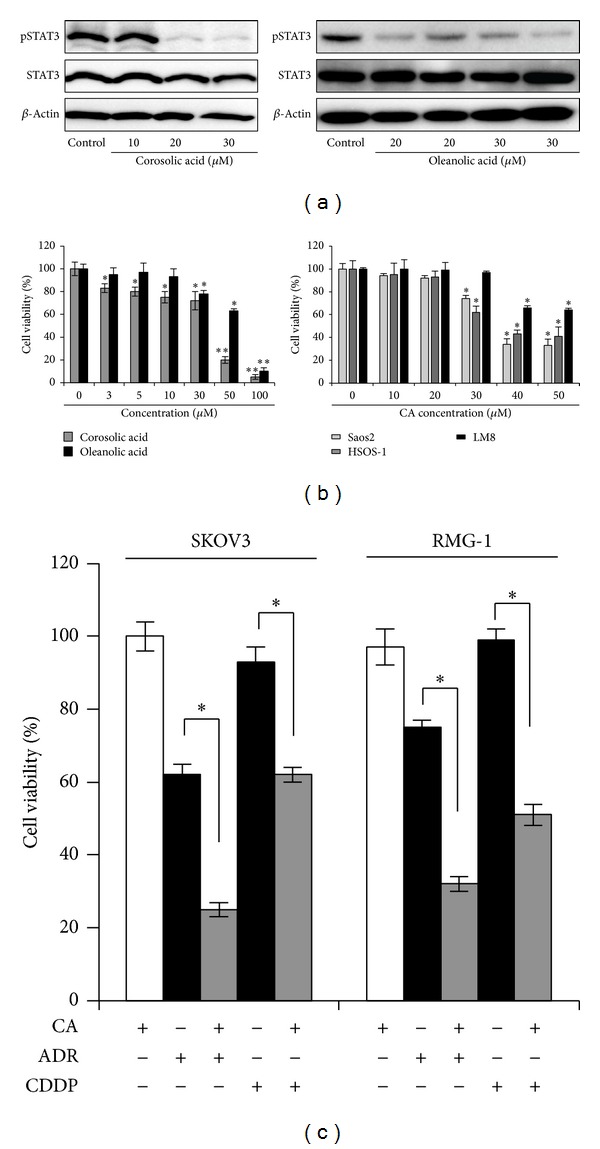
Effects of corosolic acid and oleanolic acid on STAT3 activation and cell proliferation in the tumor cells. U373 cells were incubated with the indicated concentrations of corosolic acid or oleanolic acid for three hours, after which the phosphorylated STAT3, STAT3, and *β*-actin expression was determined using a Western blot analysis (a). Glioblastoma cells (U373 cells) and osteosarcoma cells (Saos2 cells, HSOS-1 cells, and LM8 cells) were incubated with the indicated concentrations of corosolic acid and oleanolic acid for 24 hours, after which the degree of cell proliferation was determined using a WST-8 assay (b). Ovarian carcinoma cells, SKOV3 and RMG-1 cells, were incubated with 10 *μ*M of anticancer drugs (ADR: adriamycin; CDDP: cisplatin) during incubation with or without corosolic acid (20 *μ*M) for 24 hours, after which the cell viability was determined using a WST-8 assay (c). The data are presented as the mean ± SD. *P < 0.05, **P < 0.001 versus control.

CA and OA have been reported to induce apoptosis in human cancer cells, such as cervix adenocarcinoma cells, gastric cancer cells, breast cancer cells, and hepatocellular carcinoma cells [34–39]. It is clear that activation of STAT3 is critically involved in tumorigenesis [40, 41]. Therefore, we next investigated the effects of CA and OA on STAT3 activation in tumor cells. As shown in Figure 2(a), STAT3 was consistently activated in U373 glioblastoma cells. Under the assay conditions, CA and OA significantly inhibited STAT3 activation (Figure 2(a)). Furthermore, CA and OA significantly suppressed the proliferation of U373, Saos2 (osteosarcoma), HSOS-1 (osteosarcoma), and LM8 (murine sarcoma) in a dose-dependent manner (Figure 2(b)). Similar antitumor effects were observed in other malignant tumor cells, including ovarian cancer cells (data not shown), whereas these compounds did not affect cell survival in the HMDMs (Figure 1(d)), thus suggesting that effective concentrations of these compounds (30 *μ*M~100 *μ*M) do not affect normal cell viability with respect to tumor cell death. These data indicate that CA and OA suppress tumor cell proliferation by inhibiting STAT3 activation.

## 5. Synergistic Antitumor Effects of CA and Chemotherapeutic Agents

We next measured the combined effects of CA and chemotherapeutic agents on tumor cell proliferation using tumor cell lines. In this experiment, CA was used at a concentration of 20 *μ*M, as this dose has been shown to suppress STAT3 activation but not inhibit tumor cell viability (Figure 2(b)). Consequently, CA significantly increased the antitumor effects of adriamycin (ADR) and cisplatin (CDDP) in ovarian cancer cells (Figure 2(c)). Similar results were observed in osteosarcoma and glioblastoma cells (data not shown). These data suggest that CA suppresses tumor proliferation and is a potential candidate agent for enhancing anticancer chemotherapeutic agents in several types of tumor cells.

## 6. CA Suppresses Subcutaneous Tumor Development and Lung Metastasis

**Figure 3:**
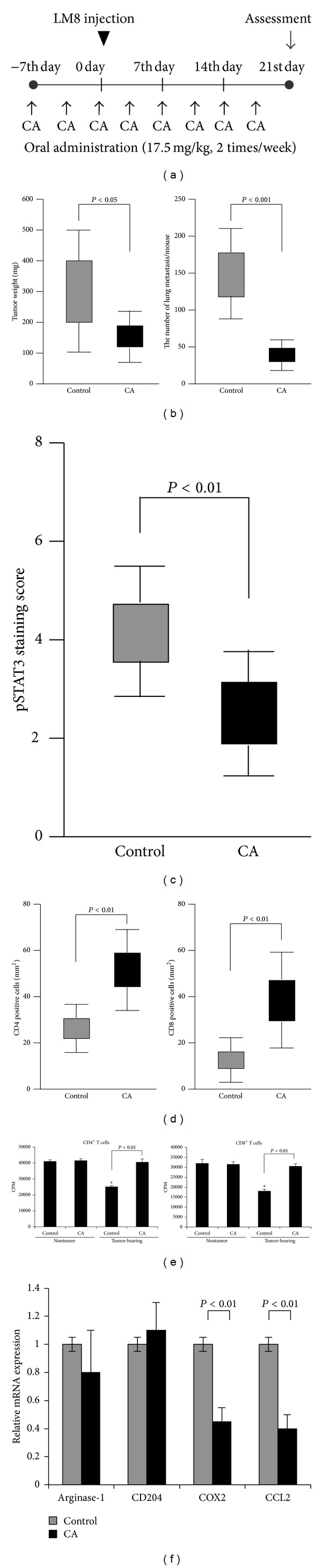
Antitumor effects of corosolic acid in the LM8-injected mice model. Protocol for administering corosolic acid (CA) and implanting LM8 (a). Effects of corosolic acid (CA) on subcutaneous tumor development and lung metastasis (b). The degree of STAT3 activation in the subcutaneous tumor tissues was evaluated using immunostaining (c). The number of CD4^+^ lymphocytes and CD8^+^ lymphocytes in the subcutaneous tumor tissues was evaluated using immunostaining (d). Inhibitory effects of corosolic acid on the MDSC-induced immunosuppressive activity in CD4 and CD8 T cells (e). MDSCs were isolated from tumor-bearing mice treated with or without corosolic acid, and the mRNA expression was examined using real-time PCR (f). The data are presented as the mean ± SD. *P < 0.05 versus control.

In the present study, the antitumor effects of CA were tested in a mouse sarcoma model. CA was administered orally before and after subcutaneous implantation with LM8 cells in C3H mice (Figure 3(a)). It is previously reported that CA was detected in blood after oral administration of CA to animal model [42]. On day 21 after tumor implantation, subcutaneous tumor development and small metastatic lesions were detected in all control mice. The results of the experiment showed that CA administration significantly suppressed subcutaneous tumor development (Figure 3(b)). In addition, CA significantly suppressed lung metastasis (Figure 3(b)). Furthermore, STAT3 activation in developed tumor cells was decreased by the administration of CA (Figure 3(c)), thus suggesting that CA suppresses STAT3 activation in both* in vitro* and* in vivo* models. On the other hand, PCNA labeling was not affected by treatment with CA (data not shown). Infiltration of immune cells and the presence of angiogenesis were evaluated using immunostaining. The results of immunostaining showed that the levels of CD4^+^ and CD8^+^ lymphocytes in the subcutaneous tumor tissues were increased by the administration of CA (Figure 3(d)). There were no significant differences in the number of CD68^+^ TAMs, CD204^+^ TAMs, or CD31^+^ vessels (data not shown). These data suggest that CA impairs tumor development and lung metastasis by activating antitumor immunity.

Inhibitory effect of CD163 expression was not investigated in this tumor model, because no CD163^+^ cells were detected in the tumor tissues of either the vehicle or CA administration group (data not shown). In our observation, the born marrow-derived macrophages hardly express CD163 at all (unpublished data). TAMs in murine tumor models are mainly from bone marrow and this might be a reason that no CD163 expression is detected in TAMs in murine tumor model.

Recent studies have demonstrated that myeloid cells are associated with systemic immune suppression in tumor-bearing hosts. In particular, myeloid cells positive for Gr-1 and CD11b in tumor-bearing mice are called myeloid-derived suppressor cells (MDSCs) due to their suppressive effects on T-cell activation. In murine tumor models, MDSCs in tumor tissues, as well as the spleen, liver, and bone marrow, have become the focus of research in recent years due to their immunosuppressive functions [43–45]. Since MDSCs and TAMs are considered to be from the same lineage, we hypothesized that CA also inhibits the immunosuppressive function of MDSCs. Initially, the number of MDSCs was evaluated using flow cytometry; however, no significant changes were observed between the control and CA-treated groups in either the spleen or bone marrow (data not shown). In order to test the immunosuppressive activity of MDSCs by means of an* ex vivo* analysis, CD4^+^ or CD8^+^ lymphocytes isolated from naive C3H mice were cocultured with MDSCs purified from the spleens of tumor-bearing or control mice. The MDSCs derived from the tumor-bearing mice significantly inhibited lymphocyte proliferation (Figure 3(e)). However, this suppressive effect was not observed among the MDSCs derived from the spleens of the tumor-bearing mice treated with CA (Figure 3(e)). These data indicate that although CA did not affect the number of MDSCs, it reversed the immunosuppressive activity of these cells. In order to investigate which immunosuppressive molecules are changed by CA administration, the mRNA expression of various MDSCs -related molecules was evaluated using real-time PCR. The results showed that the administration of CA resulted in the downregulation of cyclooxygenase-2 and CCL2 in the MDSCs (Figure 3(f)). Furthermore, CA treatment appeared to inhibit STAT3 activation in splenic MDSCs in the tumor-bearing mice (data not shown). These results suggest that CA activates antitumor immune reactions by inhibiting the immunosuppressive activity of MDSCs.

## 7. Antitumor Effects of Ursolic Acid (UA)

Although ursolic acid (UA) was not contained in the selected 200 purified natural compounds, UA is a well-known natural compound belonging to the triterpenoid family and there are many reports regarding the antitumor effects of UA [46–52]. It has also been reported that UA inhibits tumor cell proliferation in several tumor cells, such as breast cancer cells [46], gastric cancer cells [47], colon cancer cells [48], skin cancer cells [49], leukemia cells [50], lung cancer cells [51], and pancreatic cancer cells [52]. Furthermore, it has also been demonstrated that UA suppresses the growth of colon cancer cells by targeting STAT3 [48], whereas the effects of UA on macrophage activation are unknown. However, the fact that UA abrogates STAT3 activation suggests that UA impairs tumor development not only due to its direct cytotoxicity to tumor cells but also by inhibiting the protumoral functions of TAMs and MDSCs.

## 8. Tumor-Associated Macrophages as Potential Targets of Existing Medicines

There are several lines of evidence supporting the potential for targeting TAMs using existing medicines [53–55]. Bisphosphonates (BPs), such as zoledronic acid, which are antiresorptive agents approved for the treatment of skeletal complications associated with metastatic breast and prostate cancer, decrease tumor growth and lung metastasis, while zoledronic acid reverses the polarity of TAMs from M2 to M1, thus suggesting that TAMs are potential targets of bisphosphonate therapy [55]. Cyclosporine A, an immunosuppressive agent, reduces tumor growth and inhibits the alternative activations of tumor-infiltrating microglia/macrophages in a glioma model, suggesting that blocking the infiltration of microglia/macrophages and their proinvasive functions is a potential novel therapeutic strategy in patients with glioma [53]. Furthermore, trabectedin, an anticancer drug, suppresses both tumor development and TAM activation in cases of myxoid liposarcoma [54]. These reports demonstrate that the regulation of macrophage activation is a potential target for anticancer therapy.

## 9. Conclusion

**Figure 4:**
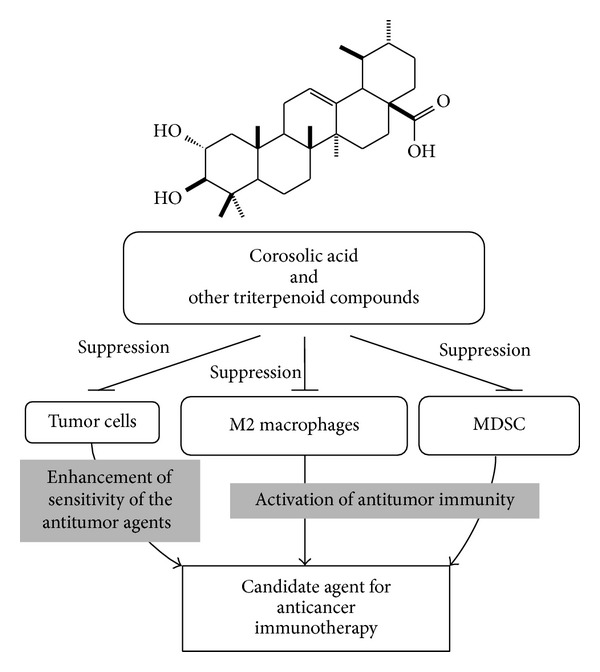
Possible mechanism underlying the inhibitory effects of corosolic acid and other triterpenoid compounds on tumor proliferation.

STAT3 is associated with tumor progression in many malignant tumors. STAT3 is considered to be an important target molecule for anticancer therapy, and many researchers have thus far reported the importance of various STAT3 inhibitors in the setting of anticancer therapy [56]. Natural compounds, such as CA and OA, exert inhibitory effects on STAT3 activation in macrophages, MDSCs, and tumor cells [29, 57, 58]. In the present study, we revealed that those compounds inhibit tumor proliferation and differentiation of macrophages toward M2 phenotype via inhibiting STAT3 activation, whereas inhibitory mechanism of those compounds on MDSCs function has not been unclear. Furthermore, our findings indicate that these compounds may be useful in anticancer therapy by targeting the immunosuppressive activity of MDSCs and TAMs via synergistic effects with anticancer agents (Figure 4). Triterpenoid compounds regulating the activation of myeloid cells, including MDSCs and TAMs, are potential candidate agents for anticancer therapy.
